# A conceptual analysis of older adults’ empowerment in contemporary japanese culture

**DOI:** 10.1186/s12877-021-02631-x

**Published:** 2021-12-01

**Authors:** Yoshihito Tsubouchi, Kyosuke Yorozuya, Akiyoshi Tainosyo, Yasuo Naito

**Affiliations:** 1 Faculty of health sciences, Naragakuen University, Nara, Japan; 2 Graduate School of Comprehensive Rehabilitation, Osaka Prefecture University, Habikino, Japan; 3 Faculty of Care and Rehabilitation, Seijoh University, Tokai, Japan; 4 Department of rehabilitation, Akitsukounoike Hospital, Gose, Japan

**Keywords:** Empowerment, Japan, Older adults, Conceptual analysis, Self-determination, Aging, Collectivism

## Abstract

**Background:**

Empowerment among older adults is a key concept for improving their health. In contrast, empowerment evolves according to cultural and historical contexts and needs to be consistently tested and constructed. The purpose of this study was to clarify the components of older adults’ empowerment in contemporary Japan and to reconstruct the definition of empowerment.

**Methods:**

A conceptual analysis was performed using Rodgers’ evolutionary method. The data sources were PubMed, Cumulative Index to Nursing and Allied Health Literature, Web of Science, Cochrane Library, and Igaku Chuo Zasshi. The search keywords were “empowerment,” “older adults,” and “Japan/Japanese.” Of the 8811 articles published between 2000 and 2019 that focused on older adults’ empowerment, we selected 60 articles that met our objectives.

**Results:**

Seven antecedents, six attributes, and seven consequences were identified. Older adults’ empowerment in contemporary Japan was defined as “the series of processes in which disclosing oneself, not only verbally but also nonverbally (e.g., through work, roles, and collaborative activities), in connection with others, objectively perceiving one’s existence and challenges, taking proactive actions based on decision-making, and utilizing one’s strengths in new work and community life.”

**Conclusions:**

This concept is useful in practice, education, and research on community development and providing support for older adults based on self-help and mutual aid, not only in Japan but also for the global aging society.

**Supplementary Information:**

The online version contains supplementary material available at 10.1186/s12877-021-02631-x.

## Background

In 2019, approximately 9% of the global population was aged 65 years and older, and this statistic is projected to reach 12% in 2030 and 16% in 2050 [1]. Specifically, East and Southeast Asia will experience the most rapid population aging, with the proportion of people aged 65 years and older rising from 11% to 2019 to 24% in 2050. Additionally, the potential support ratio in Japan was 1.8 in 2019, representing the lowest among countries and regions with a population of 90,000 or more [2]. This is defined as the number of people of working age (25 to 64 years) per older person aged 65 and older. The low birthrate and aging population key affect the labor market and economic activities. Therefore, it is necessary for countries with aging populations worldwide to focus on Japan‘s efforts to manage the pressure on public systems and finances, including healthcare, pensions, and social security systems for older adults to identify issues and develop preventive measures [2, 3].

In Japan, the prevention of confinement and frailty among the elderly has become a crucial issue in recent years [4]. To address this, Japan has established the “Outline of Measures for an Aging Society” based on the Basic Law on Measures for an Aging Society; and to promote measures for employment and income, health and welfare, learning and social participation, and living conditions [5]. The basic principle of the plan was “to create an ageless society in which people of all ages can play an active role,” which would be achieved “by reviewing the classification of people according to age and by optimizing their motivations and abilities according to their wishes.” The plan also includes “improving the community infrastructure, and creating a local community in which people at any stage of life can concretely envision their life in old age.” In conventional Japan, older adults were prohibited from living independently in their own homes. In the past, the cultural ideal was for older adults to “relax and let the younger generation take over,” and older adults with illnesses were cared for by family members, with their resting and convalescing being prioritized [6]. Since 2000 (defined as modern Japan), the number of households headed by older couples and older individuals living alone has increased, posing a challenge to traditional Japanese culture where children support their parents [6]. However, older adults cannot alter their traditional cultural thinking and remain isolated or dependent on public support [6]. This will inevitably lead to an increase in medical and nursing care costs and a shortage of human resources to provide care.

The World Health Organization Ottawa Charter for Health Promotion defines health promotion as “the process of enabling people to increase control over, and to improve, their health.” It emphasizes the need for an empowerment-based approach to improving individual skills and strengthening community activities [7]. The concept of empowerment was derived in the 17th century from the legal term “to give official authority or legal power.” Since the 1980 s, it has been used in various fields, including public health, welfare, and mental health and has been studied in different populations [8]. The definition of empowerment is applied across different contexts depending on the historical background and national culture. A predominantly used definition worldwide is Wallerstein’s description: “the process of social action that promotes the participation of people, organizations, and communities to take control of their own lives in the community and wider society” [9]. Moreover, Gibson defined empowerment as the “process of helping people assert control over factors that affect their lives,” [10] which is often used in relation to older adults. Rodwell explained empowerment as “a process that supports partnerships that respect self and others, equal decision making, and freedom to accept choices and responsibilities” [11]. Gutierrez addressed the concept expansion by contending that “the concept of empowerment has been interpreted by researchers in a variety of ways, including objectives, processes, and methods of intervention, and has been disseminated with a lack of clarity” [12]. Contrarily, Zimmerman considers empowerment as “a concept that changes fluidly according to differences in culture, perspective, and historical background” and calls for society to reconstruct the concept of empowerment according to the time and culture [13].

In Japan, Nojima introduced empowerment as “an important concept that promotes a paradigm shift in which care recipients and care providers must form a partnership and collaborate to solve problems, to demonstrate the individual’s power, while respecting the rights and autonomy of the care recipient” [14]. However, Tomoyama and Hoshi noted that by 2000, there were only 31 research papers in Japan using empowerment as a keyword [8]. Moreover, they argued that the concept of empowerment was incompatible with Japan’s unique traditions and culture, such as degree of patriarchy, not challenging superiors, and prioritizing cooperation over rights [8]. After 2000, social and disease structures gradually changed, the awareness of rights was recognized, and the measures emphasizing health promotion were strengthened.

In the field of health and medicine, the concept of empowerment has been applied to patients and the families of patients with diseases, such as diabetes, mental health illnesses, and lung cancer [15]. The application of empowerment is also increasing among older adults, especially at the community level. However, as in other countries, the definitions and interpretation of empowerment lacks consensus across researchers. Ward and colleagues excluded Japan from the analysis of empowerment research targeting Pacific Rim countries because “the concept of empowerment still does not apply to the culture of older adults in Japan” [16]. Owing to the rapidly aging Japanese population, it is imperative to reconstruct the concept of empowerment among older adults within a contemporary cultural context to develop support aimed at improving the health of older adults. In addition, it is useful to organize and disseminate the Japanese concept of empowerment to support health promotion among older adults in other countries facing an aging population.

Therefore, this study reconstructed the concept of empowerment among older adults in contemporary Japanese culture. This was achieved through reviewing research related to older adults’ empowerment in Japan and extracting and organizing a wide range of applications of the concept, including definitions, antecedent requirements, attributes, and consequences. We also examined the use of the empowerment concept in the field of medical and nursing care for older adults.

## Methods

### Concept analysis

The aim of concept analysis was to clarify the conceptual definitions of various phenomena and terms that exist in actual medical and nursing practice. The primary concept analysis methods were formulated by Walker and Avant [17], which centers around essentialism, and Rodgers and Knafl [18], which entails an innovative and evolutionary method. The latter focuses on the usage and context of a concept to clarify the definition of the target concept by elucidating not only its attributes, but also its antecedent requirements and consequences. The concept of empowerment has evolved both domestically and internationally, depending on the time period and the subject. We thus deemed Rodgers’ concept analysis method as the most appropriate method for our research on older adults in contemporary Japan; moreover, it has been widely adopted in Japan and is effective regarding practicality. This study was conducted with reference to the Preferred Reporting Items for Systematic reviews and Meta-Analyses extension for Scoping Reviews and was approved by Osaka Prefectural University Research Ethics Committee and Naragakuen University Research Ethics Review Committee.

### Search methods

According to Rodgers and Knafl’s method of concept analysis, a minimum of 30 articles from relevant academic fields (in this case, health and welfare), or at least 20% of the total, should be selected [18]. The literature search was conducted using five electronic databases that are widely used in the fields of health and welfare and are considered to have a high potential for providing practical literature. These were PubMed, Cumulative Index to Nursing and Allied Health Literature, Web of Science, Cochrane Library, and the Japan Medical Abstracts Society’s “Igaku Chuo Zasshi” web version 5.

### Inclusion and exclusion criteria

#### Inclusion criteria

1. The article was published between 2000 and 2019.
2. Eligible studies included original papers, review articles, and practical studies.
3. The article’s theme was empowerment.
4. The research participants were older adults (aged ≥ 65 years).
5. The article was written in English or Japanese.

#### Exclusion criteria

1. The target population was non-Japanese.
2. There were few or no descriptions of empowerment.
3. The participants were healthcare professionals or family caregivers.

### Search strategy

One investigator (Y.T.) extracted search terms from Medical Subject Headings and existing literature.

### Search terms

The search terms included: (empower* OR empowerment* OR patient empowerment* OR participation*) AND (aged* OR elder people* OR elderly person* OR elderly patient* OR older people* OR older adults* OR old person*) AND (Japan* OR Japanese*).

### Selection process (2000–2019)

**Fig. 1:**
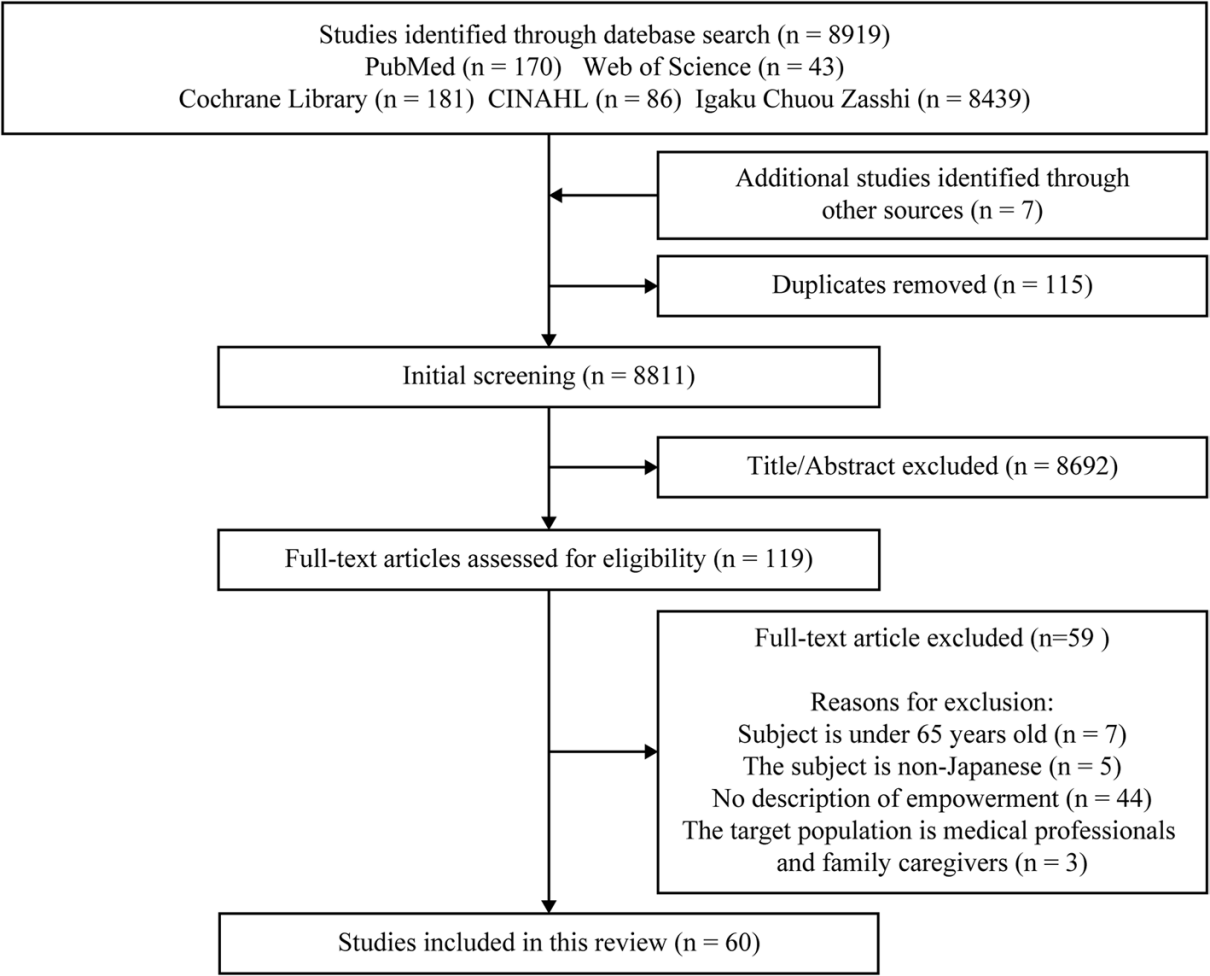
Literature search and study selection process. This shows the collected papers and the reasons for rejection

Literature search and study selection process is shown in Fig. 1.

#### Primary screening

Two investigators (Y.T. and KY) independently selected possible papers from the titles and abstracts. Any differences of opinion between the two investigators were resolved by deliberation. If necessary, another investigator (Y.N.) decided what paper to accept.

#### Secondary screening

Two investigators (Y.T. and A.T.) independently extracted papers that met the criteria from those selected in the primary screening. The selected papers were reviewed, and the accepted papers were chosen. Any disagreements between the two investigators were resolved through deliberation. If necessary, another investigator (Y.N.) decided what paper to accept.

### Data analysis

We extracted descriptions of the antecedents that precede empowerment, the attributes that constitute empowerment, and the consequences that follow empowerment for each literature. First, we carefully read each of the documents to be analyzed to clarify the overall outline. The relevant parts of the antecedents, attributes, and consequences were extracted from the raw data and written on a coding sheet for each target literature. Three researchers (Y.T., KY, and A.T.) independently extracted definitions and antecedents, attributes, and consequences for the data extraction. Any disagreements among the three researchers were resolved through deliberation. If necessary, another investigator (Y.N.) was also included in the deliberation. In extracting the data, we diligently extracted relevant items for both empowerment and disempowerment by reading the context before and after the terms in detail, focusing on the content of the concepts and the relationship between them. Then, each extracted semantic unit was labeled, coded, and categorized based on similarities and differences. Concerning attributes, for example, “talking to staff about feelings and distress over dietary management (restrictions)” and “revealing daily life problems and disputes with caregivers to nurses” were named as subcategories of “verbal disclosure” because they conveyed their thoughts and feelings in their own words. Furthermore, “an older patient with dementia participated in a chorus and clapped hands with a smile for the other participants” and “a cancer patient who refused treatment because of the lack of hope for a cure continued to fold paper cranes in his room” were considered to express thoughts and feelings that cannot be expressed in words through attitudes and actions and were named “non-verbal disclosure” as subcategories. Both “verbal disclosure” and “non-verbal disclosure” are acts of communicating one’s thoughts, feelings, and distress, and are important attributes of empowerment. Thus, these were concluded as “self-explanation.” Similarly, “an older patient became more willing to treat diabetes” and “cancer patients became hopeful about their future lives and could make self-selections” both represent inner enhancement; thus, we named them “mental empowerment” as a subcategory. “Anxiety about community life after discharge (for patients with mental illness) was reduced” and “discussing dialysis with doctors and family members reduced fear and anxiety about treatment” both represent a reduction in anxiety and were named “relief from anxiety” as subcategories. The subcategories “mental empowerment” and “relief from anxiety” have a wide range of positive psychological and psychiatric effects, and we concluded that the category “psychological stability” was the most appropriate. Four investigators (Y.T., K.Y., A.T., and Y.N.) discussed and created a table of attributes, antecedents, and consequences.

In reconstructing the definitions, the empowerment attributes from the analysis were used to create the sentences, considering the relationship and order of each category. Once created, the definition text was examined by four investigators for similarities and differences against the definitions cited in previous studies used in the conceptual analysis. In addition, the characteristics1 of empowerment of older adults in Japan were intensively discussed, and we endeavored to include them in the redefinition. Notably, all four investigators had over 10 years of experience in medical care facilities and community welfare for older adults, and three were experienced in gerontological research in Japan.

## Results

From the databases, 8811 abstracts were extracted, and 60 were included in the final analysis (Fig. 1) [19–78]. Of these, the definition of empowerment was presented in 38 cases. The definitions and citations are shown in Additional file 1. Seven antecedent requirements, six attributes, and seven consequences were identified (see Additional files 2, 3 and 4). The definition of empowerment of older adults in contemporary Japan was thus reconstructed:

The series of processes in which disclosing oneself, not only verbally but also nonverbally (e.g., through work, roles, and collaborative activities), in connections with others, objectively perceiving one’s existence and challenges, taking proactive actions based on decision-making, and utilizing one’s strengths in new work and community life.

### Antecedents

Seven categories of conditions and states were identified as being conducive antecedents for empowerment (see Additional file 2).

#### Physical and psychological characteristics of the individual

The individuals physical and psychological characteristics comprised two subcategories—*age-related diseases, decline in physical and psychological functions* and *willingness to maintain and improve health*—that describe the state of chronic diseases, incurable diseases, and decline in physical and psychological functions because of aging (i.e., negative aspects of health), and the willingness and effort taken to stay healthy (i.e., positive aspects of health).

#### Awareness of one’s own abilities and condition

This antecedent comprised three subcategories: *anxiety and/or lack of confidence*; *negative perceptions of illness, disability, and treatment*; and *self-affirmation and expectations*. These factors represented the fluctuating perceptions (negative/positive) of one’s condition, abilities, and related information triggered by intractable illness, prolonged treatment, and loss of roles because of aging.

#### Relationships with others (family, supporters) and the community

The subcategory of relationships with others included three subcategories: *feelings of failure in interpersonal relationships; isolation and loneliness*; *positive relationships with others and the community*; and *awareness of tradition and culture*. These were personal perceptions (negative/positive) of the state of relationships with others occurring in their traditional, cultural, and regional contexts.

#### Connections to personal work

Connections to personal work had two subcategories, *aspiration and commitment to the occupation* and *expectations of future occupation*. These subcategories delineated individuals’ connections to meaningful work and their hopes and expectations for future occupations.

#### Proactive behavior

Proactive behavior consisted of three subcategories: *lack of initiative and action*, *understanding relevant information*, and *action on self-tasks*. They described a state of initiative and positivity (negative/positive) in gathering information and acting on personal issues.

#### Collaboration and interaction through the human environment

This category included three subcategories: *guarantee for expression of will and self-selection*, *intervention support system*, and *quality and methods of medical and nursing care provided*. It entailed the state of the human environment surrounding older adults, including decision-making support from others, the state of communication, and the quality of medical and nursing care provided (positive).

#### Community support system and environment

The community support system and environment category included two subcategories: *community environment to prevent isolation and promote activities*, and *supportive environment from the government and related organizations*. This category represented the systems and environment (positive) of the entire community, including local efforts to prevent isolation and promote activities among older adults, and support from the government and related organizations.

### Attribution

Six categories were identified as the main words and characteristics that described empowerment (see Additional file 3).

#### Self-explanation

This attribution category consisted of two subcategories—*verbal disclosure* and *non-verbal disclosure*. The category described the disclosure of one’s inner self to oneself and others through various methods, including language, work, and facial expressions.

### Objectification of oneself and awareness of the problem

Objectification of oneself and awareness of the problem included two subcategories: *strengthening self-awareness through self-reflection* and *objective evaluation and recognition of information*. It represented the process of subjectively and objectively perceiving oneself through self-reflection and reaffirming one’s responsibility and existence as an older person or patient.

#### Gathering necessary information and self-care behaviors

This category contained two subcategories: *internal development and psychological growth* and *acquiring technical and practical skills*, which delineated the information gathering process and engagement in technological innovation to enhance and harness one’s abilities to solve life challenges.

#### Hope for the future based on decision-making

The category included two subcategories: *positive thinking* and *decision-making process*. These subcategories involved the process of adopting a positive life perspective and engaging in (shared) self-selection and self-determination based on one’s responsibility for all events related to oneself, even when facing unavoidable conditions such as old age and illness.

#### Practice proactive behaviors

There were two subcategories for practice proactive behaviors, including *preparation for behavioral practices* and *practice and continuation of actions*. They described the process of believing in one’s potential and establishing motivation for actions and activities to perpetuate positive behaviors that enrich community life.

#### Interaction with others and the community

The two subcategories were *interacting with other individuals* and *interaction with the community*, which represented how older adults (patients) shared their activities and feelings with others in their daily lives. Moreover, they expanded their collaborative relationships from personal relationships to organizations and the communities at large.

### Consequences

Seven categories were identified as secondary changes and benefits for older adults, resulting from empowerment (see Additional file 4).

#### Stability of personal health

Stability of personal health included two subcategories, *improvement of physical and psychological functions, activities* and *improvement of health status and healthy life expectancy*. They indicated that the participants had improved their physical and psychological functioning, body structure, health (lifestyle-related diseases and mental illness), disease prevention, and healthy life expectancy.

#### Psychological stability

Psychological stability comprised two subcategories: *relief from anxiety* and *mental empowerment*. The category demonstrated that acquiring accurate knowledge related to oneself reduced psychological burden (vague anxiety) and activated mental and psychological functions.

#### Acquisition of life skills and self-management

This category contained two subcategories: *resolution of targeted issues* and *acquiring social skills*. They indicated that older adults could live in the community independently and with vitality by recovering and acquiring self-management (physical condition management) abilities and skills regarding their medical care and social participation.

#### Expectations for new occupation

Older adults (with or without disease) found fulfillment and meaning in new tasks, reigniting their hopes for the future and their desire to continue to engage in community activities and social participation.

#### Behavior modification

Behavior modification included two subcategories: *internal transformation* and *behavioral changes*. This category showed that even in old age and in illness, recognizing and fulfilling one’s potential caused a shift in consciousness and encouraged continued behavioral changes through planning, execution, and reflection.

#### Regional expansion

Regional expansion comprised two subcategories: *trust and collaboration with those involved* and *contribution to regional revitalization*. The category indicated that having trust in those involved and the expansion of collaboration facilitated interaction with organizations and the region, enabling older adults to contribute to regional revitalization.

#### Social and economic effects

Individual empowerment contributed to improving the sense of well-being and health, not only for the individual but also for their family, community, and society. Community empowerment created socioeconomic benefits.

## Discussion

### The concept of older adults’ empowerment in contemporary Japanese culture

A conceptual analysis of older adults’ empowerment in contemporary Japanese culture revealed seven categories of physical and psychological characteristics of the individual. The results suggest that the conditions for empowerment are influenced by an individual’s self-perception of positive and negative situations and abilities, an individual’s behavior during interactions and working with others, and the social and physical support and environment. Gibson defines the antecedents of empowerment as: (1) the individual is fundamentally responsible for their health, and that health belongs to the individual; (2) the individual’s capacity for growth and self-determination must be respected; (3) the conditions for empowerment include mutual respect, participation, and collaboration between the healthcare provider and the client; (4) trust is essential for the empowerment process; (5) healthcare providers must empower themselves and cannot empower others; and (6) healthcare professionals must abandon the desire to control the patient and prioritize their needs [10]. Consistent with previous research, our findings highlight the importance of positive self-awareness, trust in interactions with others and the community, and empowering oneself. However, this study also indicated the impact of personal work and activity on empowerment as a characteristic of older adults in Japan. Japanese older adults are conditioned to believe that they cling to outdated beliefs and that asserting their rights and expressing their intentions are unacceptable [6]. Thus, engaging in work and creating things may provide opportunities for them to become aware of their own issues and needs. Then they can utilize their years of experience and gain control over their actions, thereby empowering themselves.

Moreover, six categories were identified as the main concepts and characteristics that described empowerment. Zimmerman identified three elements of individual psychological empowerment: (1) intra-individual constructs, (2) inter-relational constructs, and (3) behavioral constructs [13], consistent with the present findings. We also established the importance of the process of decision-making and life control for older adults. Furthermore, our findings revealed that older adults expressed themselves through interactions with others and work engagement. By gathering information and proactive participation, older adults could recognize their tasks and roles, leading to proactive behavioral practices based on decision-making and interactions with others and the community.

The process of empowerment is explained by Kubota, based on the women’s empowerment framework by Longwe (welfare, access, conscientization, participation, and control) [79]. The first stage, welfare, involves taking action to meet basic needs. The second stage, access, is having access to a variety of power-generating resources. The third stage, conscientization, entails becoming aware of one’s situation and the role one can play. The fourth stage, participation, involves proactive solutions and participation in decision-making toward the values and goals that have been recognized. The fifth stage, control, is to create new relationships and to work with others. Specifically, the first and second stages highlight the importance of dialogue through self-disclosure, which is consistent with the characteristics we identified. For older adults in contemporary Japan, systems and environments for the realization of individual needs and access to information are being developed in medical and nursing care settings and in the community. Self-disclosure, which serves as an opportunity for empowerment, is essential for the process to progress. In addition, Japan has a tradition of collectivism and prioritizing the usefulness of others and the community [3, 80]. We found that older people desire role recognition, fulfillment of their individual potential, and proactive social participation, indicating that the third to fifth stages of the process are also crucial attributes in the empowerment of Japanese older adults.

Finally, seven categories were identified as secondary changes and/or benefits for older adults resulting from empowerment. In a review study of hospitalized older patients by Castro, empowerment positively affected the quality of care, including improved accessibility, safety, and satisfaction of patients [81]. Older adults are assumed to become better informed and empowered by being more empathetic toward healthcare providers and improving their communication skills. Our findings were consistent with extant research on hospitalization environments, showing that improvements in personal health and satisfaction and recognition of accurate information can foster new life skills and self-management abilities. In addition, good communication and information acquisition affected the expansion of the community, the quality of life of family members and neighbors, and the economy, which could be attributed to the strength of the community culture among Japanese older adults. Wallerstein contended that the process of empowerment is accompanied by a change in consciousness and a sense of connectedness as the boundaries between the self (referring to both the nurse and the client) become more permeable [9]. In other studies, the targets, methods, and processes of empowerment were discussed at three levels: individual or psychological empowerment, organizational (group) empowerment, and community empowerment. Our analysis also showed that empowerment flows from the individual’s personal growth and future expectations to the expansion and enrichment of organizations and communities and has larger socioeconomic effects. This suggests that the effects may proliferate from individuals to groups and communities as the empowerment process progresses. Japan has traditionally emphasized connectedness between neighbors and being useful in one’s community. This cultural context enables the expansion of empowerment from the individual to the community.

These results suggest that the attributes and consequences of the empowerment concept are congruent with that of previous studies and that the influence of the historical and cultural context is negligible. However, it is necessary to consider the diversity of requirements for empowerment, such as age-related diseases, lifestyles, thoughts, and social environment, depending on the era and culture. We found that Japanese older adults use non-verbal expressions (creative occupation, role-playing, and group activities) instead of conversation when disclosing their feelings, hopes, hardships, and other aspects of self. To promote the empowerment of older adults in these collectivist countries, it is vital to create an environment that allows self-disclosure not only through communication but also through non-verbal means.

### Utilization of the empowerment concept in medical and community health promotion for older adults

Our results highlighted the importance of the quality of medical and nursing care provided by healthcare professionals, the significance of communication based on the older person’s self-selection and self-determination, and the value of appropriate information provision and environmental adjustment. In traditional Japanese culture, especially among older adults, following the instructions of healthcare professionals and government officials is viewed as the ideal. However, this trend led to older people’s dependence on family members and medical providers for care and medical treatment. They, thus, became apathetic, which discouraged empowerment to continue treatment and achieve independence in life management. Labonte stated that the perception that the healthcare professional has superior knowledge promotes dependency in a patient. Thus, healthcare professionals should respect individuals’ participation and be prepared to accept that their decisions may differ from the professional’s advice [82]. Gibson also supports the perspective that the client can best identify areas for change and highlights the need for client-directed interventions to be consistent with the client’s ideals, values, and goals and to be understood and shared to some degree of risk-taking [10]. Specifically, for frail and ill older patients, it is essential for the individual and the medical care provider to critically consider the meaning of aging and illness together and for the patient to consciously and independently choose health behaviors [83]. In Japan, there has been a delay in respecting the rights and decision-making abilities of older patients compared to other countries. However, the importance of shared decision-making and treatment goals between medical professionals and patients has recently been emphasized. Holmstrom stated that person-centeredness and empowerment are complementary and that supporters must be aware of an individual’s beliefs and respect their decisions [84]. Our results also reaffirmed the importance of recognizing the positive and negative aspects of past experiences, the current context, and the future associated with choices for older adults to achieve independent living (self-care) based on decision-making. In addition, empowerment can only be achieved by the individual, and it is important for medical professionals and family members to trust the older person’s ability to make choices. They must further foster an environment where an individual can gather necessary information based on an appropriate assessment. As the initial step in support, it is necessary to create an environment and community in which older adults can feel “connected to others and the community” by providing appropriate information.

 Not only communication and community participation but also engagement in work, helped participants self-disclose. It also enhanced self-efficacy, skills development, and opportunities for community participation. Thus, healthcare professionals need to not only be close to the patients and assist them with verbal communication but also to support their work practices according to their individual abilities and strengths. Older adults have the power of experience and crystalline knowledge. In addition, occupational therapists have traditionally supported meaningful work based on shared goals with the patient. It is necessary to provide support that harnesses this expertise; assesses the patient’s meaningful work; and protects the patient’s ability to exert power through collaboration with family members, local residents, and healthcare professionals. The older individual’s ability to communicate and utilize work skills presumably activates interaction among residents and spirals upward, expanding into group and community empowerment. This inevitably creates resident-centered health promotion.

This study emphasized the importance of the concept of empowerment among older adults in Japan, regardless of whether they were hospitalized or living in the community. It was theorized that unifying the definitions and concepts would likely contribute to the future development of medical and nursing care for older adults. In addition, disseminating the results of this study to other countries facing aging populations could enhance the support for older adults and create prosperous communities, even in different cultural contexts.

### Limitations

This study had a few limitations. First, the concept of empowerment is not widespread in Japanese culture, and it was insufficiently represented in the papers we analyzed. The reason is that no scale has been developed to measure empowerment of older adults, including those in residential care, and the relationship between empowerment and personal characteristics such as age and sex has not been objectively demonstrated. Therefore, the use of the concept was diverse and included substitute words, which could result in the search strategy being inadequate. In addition, studies that insufficiently expressed or described empowerment may have led to incorrect classification. Therefore, it is difficult to establish a unified concept and definition of empowerment based solely on this study. Specifically, concepts such as “self-efficacy” and “decision-making,” viewed as attributes of empowerment in this study, have been established as distinct concepts with their own substitute terms and could have been presented as different expressions and concepts in the articles in this study because of their wide use.

Second, the characteristics of culture and life management behaviors across different individualistic/collectivist countries have been identified [85–87]. However, unlike individualistic countries, such as the USA and Europe, research on the empowerment of older people in Asian countries, which are predominantly collectivist, is underdeveloped [3, 45]. Therefore, these findings should not be compared with those from other Asian countries and generalized as a characteristic of all collectivist countries.

## Conclusions

As a result of the conceptual analysis of older adults’ empowerment in contemporary Japanese culture, seven antecedents, six attributes, and seven consequences were identified. Further, a definition of empowerment was presented: “The series of processes in which disclosing oneself, not only verbally but also nonverbally (e.g., through work, roles, and collaborative activities), in connections with others, objectively perceiving one’s existence and challenges, taking proactive actions based on decision-making, and utilizing one’s strengths in new work and community life.” The most important feature of this study was that empowerment process of older adults in Japan is not only about conversation, but also about non-verbal self-disclosure (attitude, facial expression, and work works) through work performance, roles, and group activities.

The attributes and definitions obtained in this study inform the development of a scale to measure the empowerment of older adults. We also reasoned that the prior requirements and consequences are likely to contribute to the development of medical and health promotion programs for older adults in Japan as a relevant factor to older adults’ empowerment. In the future, interventions promoting the empowerment of older adults from the perspective of healthcare professionals should be adaptable so that older adults have access to necessary information and can utilize their communication and work skills.

## Supplementary Information

**Additional file 1: TABLE A1.** List of definitions of empowerment among older adults in Japan obtained through literature search. This shows the definition of empowerment used in previous Japanese papers and the names of their authors.

**Additional file 2: TABLE A2.** Journal articles used in the categorization of the antecedents examining the concept of older adults’ empowerment in Japan. This reveals the seven antecedents conducive to empowerment identified in this study and the categorization process.

**Additional file 3: TABLE A3.** Journal articles used in the categorization of attribution to examine the concept of older adults’ empowerment in Japan. This reveals six attributes for redefining the empowerment of older adults identified in this study and the categorization process.

**Additional file 4: TABLE A4.** Journal articles used in the categorization of consequences to examine the concept of older adults’ empowerment in Japan. This reveals seven consequences, which are the secondary changes and benefits of empowerment identified in this study, and the categorization process.

## Data Availability

All data generated or analyzed during this study are included in this published article [and its supplementary information files].
